# Kañihua (*Chenopodium pallidicaule* Aellen), an ancestral Inca seed and optimal functional food and nutraceutical for the industry: Review

**DOI:** 10.1016/j.heliyon.2024.e34589

**Published:** 2024-07-14

**Authors:** Gladys Moscoso-Mujica, Ángel Mujica, Ernesto Chura, Noelia Begazo, Karin Jayo-Silva, Marcos Oliva

**Affiliations:** a Universidad Nacional Mayor de San Marcos, Research Group of Toxicological Biochemistry–Biochemistry Department, Faculty of Pharmacy and Biochemistry, Lima 1, Peru; b Postgraduate School, National University of Altiplano, Puno, Peru; c Postgraduate in Environmental Sciences, Catholic University of Santa Maria, Arequipa, Peru; d Faculty of Pharmacy and Biochemistry, National University of San Antonio Abad of Cusco, Cusco, Peru

**Keywords:** *Chenopodium pallidicaule* Aellen, Kañihua, Andean seed, Functional food, Nutraceutical

## Abstract

The Andean kañihua seed (*Chenopodium pallidicaule* Aellen) is widely used as an ancestral nutraceutical with great industrial potential and is a little-researched seed. It has high biological and nutritional value due to its protein content of 15–19 %, optimal balance of essential amino acids, essential fatty acids, mineral content, vitamins, and non-bitter saponin content. It is a potential source of peptides with different pharmacological activities such as antimicrobials, antioxidants, antihypertensives, and antidiabetics, among others. It has been a functional food in the Altiplano of Peru and Bolivia since the time of the Incas (between the 12th and 16th centuries) and is a functional food proposal for the world. In this bibliographic review, we present a detailed scientific description of the botanical characteristics, genetics, phytochemical composition, bioactives, and nutritional value. The potential uses at an industrial, medical, pharmacological, and biotechnological level and current advances in scientific research on the kañihua seed. In addition, it is an alternative grain to guarantee food security in terms of quantity, quality, and opportunity**.**

## Highlights

• Andean kañihua seeds are a novel ingredient used in foods and nutraceuticals.
• Kañihua seed has high biological and nutritional value with 15–19 % protein content.
• It is a functional food and an ancestral nutraceutical used since the Inca times in Perú and Bolivia.
• Kañihua could be an alternative that guarantees food security in the future.

## 1 Introduction

The pre-Hispanic cultures that occupied the Peruvian-Bolivian Andes domesticated various species of Andean seeds, such as kañihua (*Chenopodium pallidicaule* Aellen), through sustainable agriculture [1]. In 1911, botanist Cook, a member of the expedition that discovered the citadel of Machu Picchu in Cusco, Perú, stated that in the 16th century, there were more domesticated species in the Andes than in Asia or Africa [2,3]. Kañihua is essential for rural Andean residents to ensure their food security, nutrition, and self-sufficiency. Its cultivation and the promotion of its consumption must be based on its natural values, which form a crucial part of the traditional knowledge of rural livelihoods [4]. In 1970, Gade reported that Andean farmers domesticated wild kañihua in quinoa and potato fields. Observing that it had survived the harsh climate of the Peruvian Altiplano, Gade described it as a rustic domesticated plant that retained the characteristics of wild seed-producing plants, such as breakage and non-uniform maturation [5]. Bruno [6] reported patterns in the presence of domesticated wild kañihua seeds at archaeological sites in Lake Titicaca, Bolivia. Archaeobotanical evidence indicated that kañihua was domesticated after 250 CE, and with regional paleoclimatic evidence of frequent climatic fluctuations, they showed that this crop was a diversified supply, making it a buffer crop against climatic risks.

Andean seeds improved and preserved by the inhabitants of the Altiplano are among the most promising plant sources used in food, owing to their diversity and nutritional value. Moreover, they show an optimal balance of essential amino acids comparable to those of animal origin, attributed to their rich content of Lys (6.3 mg/16 g N_2_), one of the missing amino acids in most plant foods [7]. In addition, kañihua presents unique agronomic characteristics such as being resistant to harsh climatic conditions, including at low temperatures of up to −4 °C, strong winds, frost, and long droughts that can produce only 145 mm of annual rainfall. It grows on edaphic soils, adverse owing to salinity and clay–sand texture, and is considered a C_4_ plant because of its high efficiency in using solar radiation and fixing carbon dioxide from the atmosphere that allows it to grow between 3000 and 4200 m of altitude [1,8].

In this systematic bibliographic review, we aim to analyze, evaluate, and provide information sourced from books and scientific articles on kañihua, an Andean seed, which is little researched compared with other seeds. Kañihua represents a legacy of ancestral Inca knowledge with valuable nutritional and agronomic benefits and constitutes an ancestral nutraceutical and functional food with botanical, agronomic, and nutritional value, and great agro-industrial potential. Additionally, valuable information is available from studies on its pharmacological, functional, and nutraceutical activities. This integrated information from the agronomic, industrial, and health fields will help researchers understand, enhance, and disseminate the attributes of Andean kañihua seeds cultivated since ancient times in Perú and Bolivia. Moreover, this knowledge will also help us understand the limitations associated with its cultivation, promotion, and studies. Notably, food supply and nutritional security will be major challenges for future generations that will have to overcome abiotic productivity stress. The information presented in this review indicates that encouraging kañihua cultivation and consumption in synergy with studies could provide an alternative as a critical plant resource to improve rural, marginal, remote, and urban food systems and overcome the challenges of climate change and abiotic stress.

## 2 Methodology

A systematic review of books, scientific articles, and summaries of national and international conferences, published and available from Scopus, MedLine, PubMed, and Google Chrome Academic, was conducted in Spanish and English, without restrictions on the date and type of study.

## 3 Analysis and integration of information

### 3.1 Botanical description

**Fig. 1:**
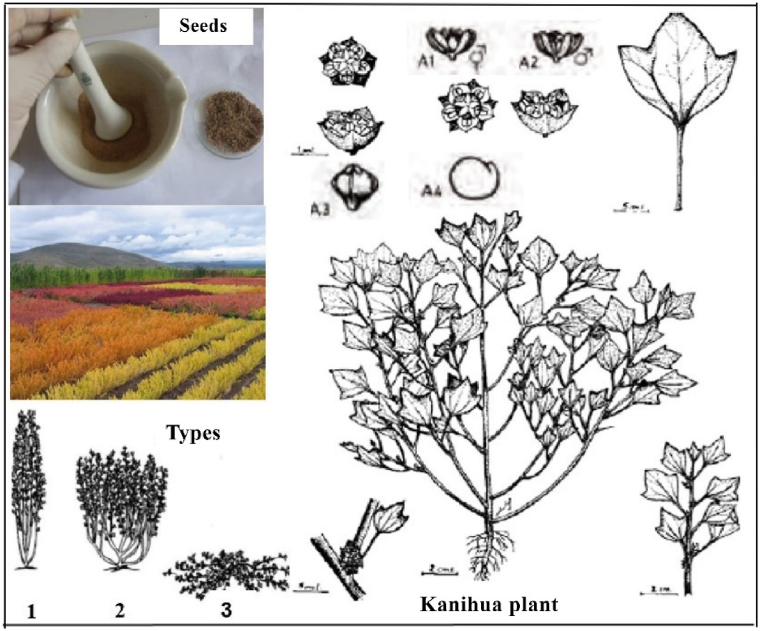
Kañihua plant (*Chenopodium pallidicaule* Aellen). Seeds (Photo: Gladys Moscoso-Mujica and Angel Mujica). Kañihua plant; A1, hermaphrodite flower; A2, flower masculine; A3, fruit; A4, seed. Types (plant growth habit): 1, Sayhua; 2, Lasta; 3, Pampa Lasta. References: Repo-Carrasco (2009) [16], Mujica (2000) [1], FAO (2000) [17], and Tapia 1997 [2].

Kañihua belongs to the *Amaranthaceae* family, an annual dicotyledonous plant, and exhibits a percentage of self-pollination between 60 and 80 %, depending on the variety, and outcrossing is greater than that in quinoa. It does not have petals and has small flowers, of which 80–90 % are hermaphroditic flowers, most have a single stamen, 10–20 % are pistillate, and 0.5 % are male-sterile, with a few flowers having three overly small stamens [9,10]. The androecium is formed by 1–3 stamens [1], and the gynoecium has a superior, unilocular ovary formed by the pistil terminated by two apical stigmatic branches, generally welded at its base [9]. The flowering time is 11.3 days, the opening of the flowers lasts 5.3 days, and flowering begins 50 days after sowing. Owing to the small size of the flowers, it is difficult to carry out manual crossings; however, crosses can occur naturally [10,11]. The leaves alternate, presenting short and thin petioles, with thickened rhomboid-shaped blades covered with vesicles and measuring 1–3 cm long, divided into three lobes at the top, with three well-marked veins on the underside that join near the apex after the insertion of the petiole. The petiole is almost uncovered, which protects the inflorescence [12,13]. The fruit is a small achene covered by a perigonium, generally gray or black, with an extremely fine episperm. The seeds do not present dormancy and can germinate on the parent plant when it has sufficient moisture. They are 1–2 mm in diameter and a little compressed, and their embryo is curved and pear-shaped [14]. The stem, in cross-section, is round or circular and is covered by pubescent vesicles, and its size varies from 25 to 70 cm based on the type of growth [13,14]. Based on the type of growth of the kañihua plant, it is classified as Sayhua, which shows upright growth; Lasta, which shows extreme branching from the base; and Pampa Lasta, which creeps [14,15], as shown in Fig. 1.

### 3.2 Genetics

It is a diploid species with 2n = 2x = 18 chromosomes, and chromosome duplication was achieved, obtaining lines of 2n = 4x = 36 chromosomes [14]. The simple characteristic of the color of the seedlings and seeds shows inheritance with allelic dominance. Red seedlings (R) and black seeds (B) were dominant over green seedlings (rr) and brown seeds (bb) [10]. Genetic diversity and variability are found in their distribution or cultivation areas and are mainly related to plant characteristics such as growth, size, seed or grain color, and the color of the stem and foliage (Table 1) [10,14,15].

**Table 1:** Variation in the characteristics of kañihua plants and seeds in terms of shape, size, and color.

Kañihua	Growth of plant	Seed size (mm)	Seed color	Stem and foliage color	References
Saiwa	Upright	1.0	White, light brown, black (Ccoito)*	Green, yellow orange	10, 14, 15.
Lasta	Much branched from the base	0.5	Black (Ccoito)*, light brown	Pink, red, purple	
Pampa Lasta	Creeping	0.5–1.0	Light brown	Green, yellow	

*Denomination of black kañihua seeds by Altiplano inhabitants.

Thirty-two varieties have been found in the kañihua germplasm collection at the National University of the Altiplano of Puno [18,19]. Currently, the main cultivated varieties are the Cupi-type Lasta, dual-purpose seeds/forage Rosada, Lasta-type Lasta, dual-purpose medium seeds, and Ramis-type Lasta. The local Peruvian ecotypes were kañihua Lasta Chilliwa (pink), Puca (red), Morada (dark), Condorsaya (gray-brown), and kañihua Saiwa Acallapi, Puca, Morado, Huanaco, and Condorsaya. The main ecotypes of the Aymara area are Choque Sillihua, Cunacutuma, Kitay llama, Chuto, Kello, Illama, Alfenica, Alverja, Airampo kañihua, and Pito, among others [14].

### 3.3 Crop and production

The botanical characteristics of kañihua allow it to resist extreme climatic conditions typical of the Peruvian-Bolivian highland area. In Perú, the area under kañihua plantation exceeded 10,000 ha until 1940, later replaced by oats and fodder barley crops. According to the report of the National Institute of Statistics and Informatics, in the agricultural campaign from 1999 to 2018, the principal producing departments of kañihua in Bolivia are La Paz, Oruro, Cochabamba, Potosi, and Tarija [20]. In Perú, according to the report of the Integrated Agricultural Statistics System, 96.0 % of the national production occurred in Puno, followed by 3.2 % in sierra part of Cusco, and 0.2 % in Arequipa, Ayacucho, Junin, and Huaraz [21]. Kañihua production in Perú in 2010 was 4366 t, corresponding to 5554 ha of the planted area, and in 2018, it was 5000 t, corresponding to approximately 7000 ha of the planted area [11,21]. Mujica et al. [11] indicated that Perú has a larger area under kañihua cultivation than that reported in Bolivia. Breeding efforts remain limited, and the seed yield per plant has not increased in the last 10 years. Another study indicated that the kañihua germplasm bank in Puno, Perú has approximately 400 accessions and that the varieties with the highest yields are INIA-406, Cupi, and Ramis. The accessions show high intra-variability, with accession 03-21-23 having the highest yield and accession 03-21-315 having the lowest. The accession that shows the highest plant height is 03-21-246, and that with the highest canopy is 03-21-246, both showing satisfactory performance. Therefore, improving the genetic stability of reported accessions was recommended [22].

The cultivation of kañihua seeds in the Andes has received little or no attention regarding the development of new varieties, and only native and revalued varieties exist. In recent decades, the production of these grains has been adversely affected by climate change, which, together with the climatic variation in the Andes, alters the regime of climatic factors and, therefore, the cultivation and production of kañihua seeds. New genetic materials, sources of characteristics, and methods are needed to improve production in the context of climate change [23]. Kañihua is an abandoned and underused grain whose flour is a suitable alternative for people with celiac disease, combats obesity, and is an antioxidant. It is organically cultivated by small farmers in Perú and Bolivia under harsh conditions typical of the Altiplano, as no pest or disease affects the development and growth of kañihua significantly; however, yields are low at *ca* 1100 kg/ha, depending on the variety and growing conditions of the crop [24]. Several studies have indicated that the crop has great potential to be grown on a larger scale than currently observed in arid areas of the Andes and possibly in other areas, as it benefits soil fertility and thus allows the sustainability of Andean agriculture. Therefore, Peruvian and Bolivian farmers conserve a large genetic pool of kañihua seeds, and the gene banks of both countries maintain their collections in an *ex-situ* state. However, this crop is facing the danger of extinction due to the growing shift toward alternative commercial crops that provide quick economic income to Andean farmers and residents [14,23,25].

### 3.4 Phytochemical composition

**Fig. 2:**
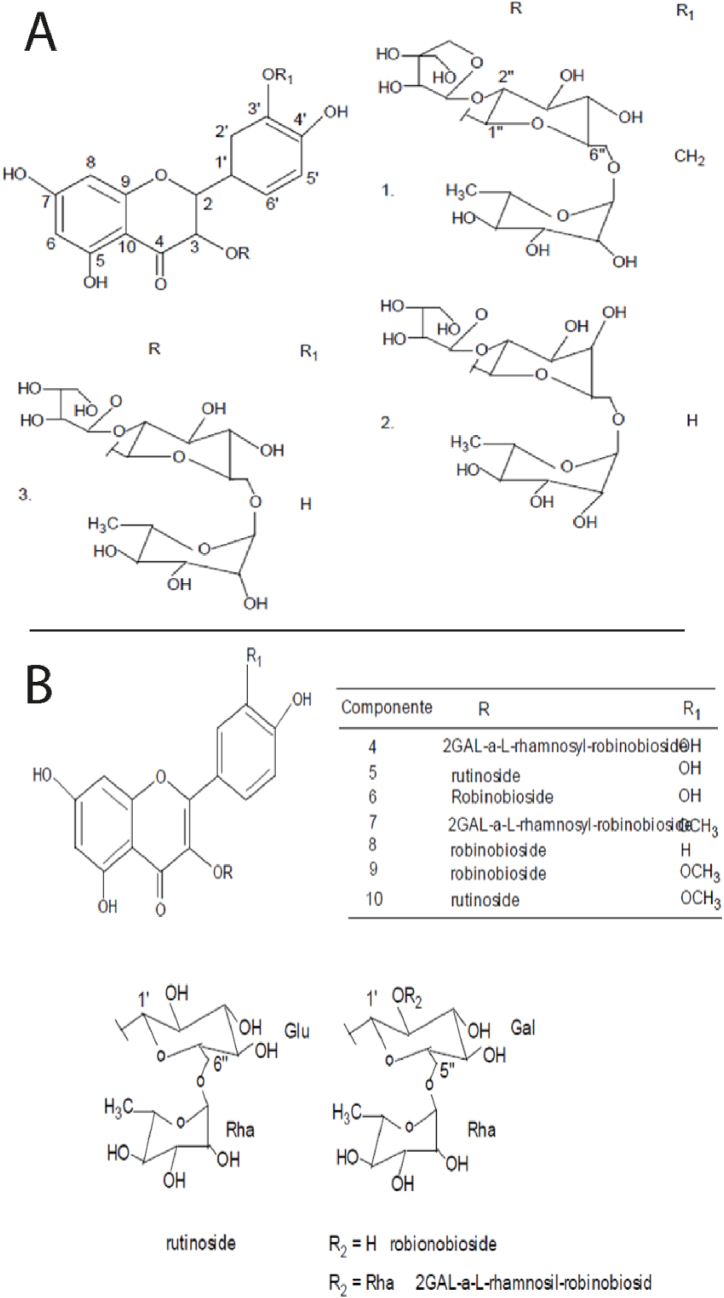
A) Chemical structures of flavonol glycosides isolated from kañihua seeds, 1) 2-GAL-α-l-rhamnosyl-robinobioside, 2) Rutinoside, 3) Robionobioside. B) Chemical structures of flavonol apiosides, 1) Isorhamnetin 3-*O*-β-d-apiofuranosyl-(1 → 2)-*O*-[α-l-rhamnopyranosyl (1 → 6)]-β-d-glucopyranoside, 2) Quercetin 3-*O*-β-d-galactopyranoside, 3) Quercetin 3-*O*-β-d-apiofuranosyl (1 → 2)-*O*-[α-l-rhamnopyranosyl (1 → 6)]-β-d-glucopyranoside. References: Rastrelli et al. (1996) [26] and Rastrelli et al. (1995) [27].

Kañihua seeds contain glycoside flavonoids, such as 2–GAL–α–l–rhamnosyl-robinobioside, rutinoside and robinobioside, flavonoid apiosides, and three types of terpenoid saponins [26,27], as shown in Fig. 2.

A study conducted by Estrada et al. [28] reported that saponins of kañihua do not have the characteristic bitter taste of quinoa and tarwi (*Lupinus mutabilis*), which favors the agro-industrial transformation of kañihua seeds for greater consumption than currently observed.

Another study reported the qualitative phytochemical analysis of kañihua seeds as containing abundant amounts of amino acids, tannins, and sugars; moderate amounts of steroids; and regular amounts of flavonoids, saponins, and phenolic compounds [29]. Repo-Carrasco and Encina [30] reported phenolic compounds such as flavonoids, phenolic acids, and polyphenols in the kañihua seed, showing a high content of total phenolic compounds of 85.71 mg/gallic acid/100 g. The high content of total phenolic compounds indicates promising bioactive characteristics such as high capacity antioxidant in the hydrophilic phase, e.g., 1509.80 μg Trolox/g, as in other foods such as blackberries with 1784.0 μg Trolox/g. Cisneros-Zevallos [31] reported that the fraction of flavonoids with free hydroxyl groups is responsible for sequestering free radicals, demonstrating the high antioxidant capacity of kañihua seeds. Peñarrieta et al. [32] showed eight compounds in kañihua seeds, including catechin, gallato, vanillic acid, kaempferol, ferulic acid, quercetin, resorcinol, and 4-methylresorcinol; thus, linoleic acid, which is a phenolic compound, acts as a flavor enhancer, particularly in roasted seed flour. The maximum content of total phenolic compounds was 71.2 μmol gallic acid equivalents/g dry weight, and the maximum total flavonoid content was 11.4 μmol catechin equivalents/g dry weight.

### 3.5 Nutritional value

#### 3.5.1 Macronutrients

Interest in forgotten or underexploited Andean crops has increased in recent years, as they are considered complete crops at the agronomic, nutritional, and biological quality levels [14]. Proximal analysis of food determines its nutritional value in terms of composition and content and is critical for ensuring quality control. Kañihua seeds are considered by the Food and Agriculture Organization of the United Nations and the World Health Organization (FAO/WHO) as a complete food because of its nutritional value and biological quality [33,34]. It has high amounts of proteins, as in the kañihua Ramis and Cupi-Sayhua varieties, with 16.2 % and 18.7 % protein content, respectively, compared with the protein content of a few cereals [33,34] (Table 2).

**Table 2:** Results of proximal analysis and a description of the amino acid content of kañihua seeds compared with that of other seeds.

Proximal analysis (%)	Seeds							References
	Kañihua	Quinoa	Kiwicha	Rice	Broad bean	Bean	Tarwi	
Proteins	16.7	22.0	12.8	9.9	23.6	22.9	48.8	22, 36, 59, 66, 67.
Moisture	11.6	10.5	11.5	12.9	12.3	12.2	8.1	
Ash	7.4	3.06	2.3	0.6	2.7	3.2	2.1	
Lipids	10.0	6.7	5.7	1.6	0.9	1.3	21.2	
Fiber	5.8	3.5	4.4	0.7	0.8	3.9	3.8	
Carbohydrates	50.9	51.1	63.3	74.2	50.3	56.7	19.7	
Crude fat	7.6	6	7.6	2.2	–	–	–	
Raw fiber fibra	6.1	4.0	5.0	10.2	–	–	–	

Amino acids (mg/16 g N_2_)	Seeds					References
	Kañihua	Quinoa	Kiwicha	Rice	Wheat	
Aspartic acid	7.9	7.8	7.4	8.0	4.7	14, 23, 66, 67.
Threonine	4.9	3.4	3.3	3.2	2.9	
Serine	3.9	3.9	5.0	4.5	4.6	
Glutamic acid	13.6	13.2	15.6	16.9	31.3	
Proline	3.2	3.4	3.4	4.0	10.4	
Glycine	5.2	5.0	7.4	4.1	6.1	
Alanine	4.1	4.1	3.6	5.2	3.5	
Valine	4.7	4.2	3.8	5.1	4.6	
Isoleucine	6.8	3.4	3.2	3.5	4.3	
Leucine	6.1	6.1	5.4	6.7	7.5	
Tyrosine	2.3	2.5	2.7	2.6	3.7	
Phenylalanine	3.5	3.7	3.7	4.8	4.9	
Lysine	5.6	5.6	5.6	2.8	3.2	
Histidine	2.7	2.7	2.4	2.2	2.0	
Arginine	8.2	8.1	8.2	6.3	4.8	
Methionine	3.0	3.1	3.8	1.3	3.6	
Cysteine	1.6	1.7	2.3	2.5	2.2	
Tryptophan	0.9	1.1	1.1	1.1	1.2	
N_2_ of seed	2.5	2.1	2.2	1.5	2.2	
Protein	15.7	12.8	13.4	9.5	14.0	

Data is expressed on a dry weight basis.

##### 3.5.1.1 Amino acids

Kañihua seeds have a balanced composition of amino acids similar to that exhibited by the casein protein of milk [35]. Mujica and Chura [14] reported that kañihua seeds have essential amino acids such as Thr, Ile, and Lys, with higher values than in seeds of relatively more widespread Andean grains such as quinoa, kiwicha, and other crops widely used worldwide, e.g., rice and wheat grains. Furthermore, Lys content stands out as a scarce amino acid in foods of plant origin; it has a significant proportion of sulfur amino acids, the same His content as quinoa, and is higher than other grains (Table 2). In addition, they have amino acid contents higher than those recommended by the FAO [7]. The amino acids present in high concentrations in kañihua include Lys, which is critical in the formation of cells, tissues and organs of the human body, i.e., it fulfills the function of proteinogenesis and allows the absorption of essential mineral nutrients; Ala as a source of energy for muscles, brain and nervous system; Gly that acts as a calming neurotransmitter in the brain regulating motor functions; and Pro that participates in the repair of muscle joints in collagen synthesis [7].

##### 3.5.1.2 Proteins

Kañihua seeds are a critical source of proteins, with high values between 15 and 19 % depending on the variety, and classified as albumin, globulin, prolamin, and glutelin [36]. There are few studies on the analysis of protein fractions extracted from kañihua, including the study conducted by Scarpati and Briceño [37], who reported 41 % albumins and globulins, 28 % prolamins, and 31 % glutelins to be present in kañihua. Moscoso-Mujica et al. [38] carried out the first fractionation of integral proteins of the kañihua seed based on their solubility. They showed that the Ramis variety contains 15, 24.1, 25.7, 9.6, and 22.9 % albumins, 7S globulins, 11S globulins, prolamins, and glutelins, respectively. Similar values were observed in the Cupi-Sayhua variety with 15.8, 26.3, 26.7, 9.9, and 21.5 %; albumins, 7S globulins, 11S globulins, prolamins, and glutelins, respectively. The content of prolamins, which constitute gluten proteins and are responsible for gluten immunotoxicity, in kañihua is relatively less; therefore, kañihua is an ideal food for patients with celiac disease. Moscoso-Mujica et al. [33] reported the total hydrolysis (h_tot_) values in mE/g of albumins, 7S globulins, 11S globulins, and glutelins extracted from the Ramis variety of kañihua as 8.2, 8.7, 8.9, and 7.8 % and the corresponding values for Cupi-Sayhua were 9.2, 9.9, 9.7, and 8.5 %, respectively. Its values helped determine the degree of hydrolysis of each protein fraction.

##### 3.5.1.3 Carbohydrates and fiber

Repo-Carrasco [39] reported that the free sugar content in kañihua seeds was higher than that in a few cereals, with a total sugar content of 6.5 % consisting of 1.8, 0.4, 1.7, and 2.6 % of glucose, fructose, maltose, and sucrose, respectively. Bock [40] reported a total fiber content of 26–27 %, soluble fiber content of 4.1–4.4 %, and insoluble fiber content of 22–24 %. Both authors reported that kañihua seed is an excellent source of dietary fiber compared to other seeds, Andean seeds, and cereals, owing to the perigonium content that covers the seed as an optimum cellulose contributor. Mujica et al. [41] reported in kañihua seeds a high amount of insoluble fiber of 3.49 g/100 g of dry–weight and insoluble fiber of 16.41 g/100 g of dry-weight, compared to quinoa with 2.49 g/100 g and 7.80 g/100 g, respectively. Fibers fulfill critical biological and nutritional functions, e.g., water retention in biological processes, sensitization to fermentation, inhibition of digestive enzymes, facilitating the binding of bile acids, reducing plasma cholesterol levels, increasing intestinal peristalsis, and body temperature control.

##### 3.5.1.4 Fatty acids

The content of unsaturated fatty acids in kañihua seeds are 72–85 %. They contain essential fatty acids, i.e., linoleic acid (C18:2, Omega 6) and linolenic acid (C18:3, Omega 3), at a concentration of 42.6 and 6.0 %, respectively [39]. Moreover, they also contain other fatty acids, such as oleic acid (C18:1, Omega 9), palmitic acid (C16:0), and stearic acid (C18:0), at a concentration of 24.3, 17.9, and 0.4 %, respectively [39]. Del Carpio et al. [42] reported an extraction yield of 2.14 ± 0.01 g of extracted oil per 100 g of kañihua dry-weight seeds, with unsaturated fatty acids being the most abundant at 42.1, 24.7, and 3.0 % contents of linoleic acid, oleic acid, linolenic acid, respectively. Furthermore, unsaturated fatty acids such as those found in kañihua seeds participate in the regulation of blood pressure, coagulation, and kidney function because they are precursors of prostaglandins, leukotrienes, and prostacyclins [14].

#### 3.5.2 Micronutrients

Kañihua seeds contains necessary micronutrients (Table 3), including calcium, magnesium, and iron, which play vital roles in various reactions in organisms and as co-factors in metabolic pathways [41,43]. They contain different vitamins, such as vitamin K [39] and tocopherol isomers with antioxidant activity that provide a greater extent of conservation to the seed [44].

**Table 3:** Mineral and vitamin content of kañihua seeds.

Mineral (mg/100g)	Amount	References
Phosphorus	375.0	37, 55, 67.
Calcium	141.0	
Iron	17.6	
Sodium	30.0	
Potassium	330.0	
Magnesium	3.36	
Sulfur	120.0	
Manganese	4.1	
Copper	3.4	
Zinc	4.55	
Selenium	0.04	

Vitamins (mg/100g)	Amount	References
Carotene	2.0	39, 55, 67.
Retinol (A)	0.22	
Thiamin (B1)	0.8	
α-Tocopherol (E)	22.0	
Riboflavin (B2)	0.7	
Acid pantothenic (B5)	0.57	
Biotin (B7)	8.0	
Folic acid (B9)	37.5	
Ascorbic acid (C)	2.2	
Niacin (B3)	1.5	

### 3.6 Reported uses of kañihua

There is little research on kañihua compared with that on cereals and other Andean seeds, such as quinoa and kiwicha. The various applications described below demonstrate the potential use of different parts of the kañihua plant, particularly seeds (Table 4). Most of the reported investigations were considered and classified as presented below.

**Table 4:** Different uses of kañihua (*Chenopodium pallidicaule* Aellen).

Typology of use	Used for/way of use	References
**Ethnomedicinal**	Convalescent patients.	1, 7, 16.
	Hypercholesterolemic.	
	Atianemic.	
	Labor problems in parturient women.	
	Dissolved in vinegar.	
	Typhoid fever.	
	Repellent against insect.	
	Arachnid bites.	
	Bone fractures.	
	Prevention of osteoporosis.	
	Prevents melancholy and sadness, tuberculosis, malnutrition.	
**Rituals**	Llipta: Kañihua + coca leaves.	12, 14, 33.
	First haircut of infants (Rutuchi).	
	Marking of animals (Uywa Ch'uwa).	
**Alimentary**	Kañihuaco (Qañiwaku), pito: energizer, altitude sickness, dysentery, qharisiri disease.	1, 14.
**Industrial**	-Bakery industries.	7, 11, 14, 37, 43, 44, 46, 47.
	Cookies, and cakes.	
	Wholemeal bread.	
	Oil industry.	
	Animal food industry.	
	Agricultural industry.	
	Food industry.	
	Kañihua starch.	
**Culinary**	"Kañihuaco" (Qañiwaku) and "pito of kañihuaco".	2, 11, 26, 48, 49.
	Soups (Qañiw soup).	
	Creams.	
	Stews.	
	Desserts.	
	Soft drinks.	
	Hot drinks.	
	Cakes.	
	K'ispiño or Q'uispiña.	
	Designer food (gourmet).	
	Novo Andean.	
	Vegan cuisine.	
	Pasta of kañihua.	
	Food formulations.	
**Functional and biotechnological foods**	Antioxidant activity.	4, 40, 64, 63, 36, 62.
	Protein fractions: Albumins, 7S globulins , 11S globulins , glutelins.	
	-Dark chocolate with kañihua.	
	Functional food for cosmetic formulations.	
**Nutraceuticals**	Antianemic activity.	31, 65, 66, 69, 70, 71, 72, 73, 74.
	Diabetes.	
	Angiotensin–I–Converting enzyme (ACE–I) inhibitors.	
	Antioxidant activity.	
	Antimicrobial peptides (PAMs).	
	Elderly-friendly kañihua food conforming to the korean industrial standard: antioxidant, and antidiabetic properties.	

#### 3.6.1 Ethnomedicinal and ritualistic uses

The wide ethnomedicinal use of kañihua has been preserved from generation to generation, allowing for the integral use of the entire plant. Ground dry seeds dissolved in water are administered to patients with convalescence, hypercholesterolemia, or anemia and parturient women with labor analgesia, and those dissolved in vinegar are administered to patients with typhoid fever [18]. Kañihuaco (Qañiwaku) is prepared by roasting clean seeds without burning them and manually grinding them using small stone mills (Kona). The product obtained is an overly fine aromatic flour, which is comforting and delicious when consumed, although the process is relatively laborious. The consumption of Kañihuaco energizes people after long trips, counteracts altitude sickness, fights dysentery, and prevents qharisiri disease [1]. Kañihua flour can be consumed by people allergic to gluten (celiacs) or those who cannot consume products made using wheat, rye, barley, or oats [7]. In addition, ash obtained from its stems can be used as a repellent against insect and arachnid bites. Traditionally, kañihua also has cultural significance. Andean residents consume the remains of the burned kañihua seeds in the form of ash and make “llipta,” a paste rich in calcium and potassium consumed (chacchado) together with coca leaves (*Erythroxylum coca*, Kuka) as a sacred leaf of the Inca culture. In rituals such as the first haircut of infants (Rutuchi) and the marking of animals (Uywa Ch'uwa), the entire kañihua plant is used. During the construction of houses, seeds are placed at the corners of foundations for good luck [12,14,35].

#### 3.6.2 Industrial uses

In recent years, there has been a growing interest in Europe, North America, Asia, and Africa in promoting and revaluing the cultivation of Andean grains, such as kañihua, considering that these species offer alternative potential in different industries. Such alternative food sources are needed to ensure food security and improved nutrition for the global population against the challenges caused by climate change and soil degradation [7,11,39]. Kañihua seeds are used in the bakery industry to make different types of wholemeal bread, cookies, and cakes; usually, toasted kañihua flour is mixed with another cereal flour in a ratio of 20:80 (w/w) [14]. Aro and Calsin [45] proposed a food mix formulation based on seed flours with adequate nutritional value and organoleptic properties, containing 28.8, 9.3, 5.0, 6.0, 3.0, 8.0, 29.5, 8.0, 2.0, 2.0, and 0.25 % of pearl quinoa, kañihua, barley, broad bean, corn, soy, sugar, vegetable oil, essence, tricalcium phosphate, and vitamins, respectively. The mix showed a high amino acid content or chemical computation (AC) of 99.74 % and a gelatinization index of 98.3 % after the extrusion cooking process at 15 % humidity, 180 °C, and a screw speed of 457 rpm. Oil extracted from kañihua seeds can be used to obtain high-quality vegetable oils for human consumption, owing to its essential oil and PUFAs content [11]. Animal food industries, such as the poultry industry, use products made using 80 % kañihua flour, fish meal, cotton paste, salts, and weeds [1,11]. In the agricultural industry, as forage for animal feed in drought-prone areas where other forage species do not grow or are unsuitable for consumption by animals such as South American camelids, the optimal time to harvest kañihua would be at 100 and 110 days of germination [46]. Mujica & Chura [14] also recommended using it as forage, harvesting around 100 days after planting. Gomez et al. [47] described kañihua as a promising superfood in the food industry because of its nutritional and functional characteristics compared to other Andean grains and cereals. They highlighted the protein quantity and balanced composition of essential amino acids and unsaturated fatty acids at high concentrations. They also described the antioxidant capacity of the grains. They highlighted that kañihua production and consumption were limited to the producing regions. Repo-Carrasco et al. [48] reported that kañihua seed processing can modify the content of phenolic compounds, kaempferol, and quercetin. Heat treatment alters the release of these compounds from the seeds, making them more bioavailable. In milling, the bran fraction must be collected and used in food products because it concentrates most bioactive compounds. Pumacahua-Ramos et al. [49] showed nano characteristics of kañihua starch extracted from the Ramis variety. The starch molecules had a polyhedral shape with a diameter ranging from 712 to 955 nm, and the superficially agglomerated starches showed low roughness. X-ray diffractometry showed characteristic peaks of type A, a relative crystallinity of 28.52 % with a transmittance of 1.33 recorded in Fourier transform infrared spectroscopy analysis. The gelatinization enthalpy and temperature were 3.64 J/g and 62.7 °C, respectively. Thus, granulated starch obtained from kañihua has potential applications in the pharmaceutical, cosmetic, chemical, and food industries.

#### 3.6.3 Culinary uses

The kañihua seeds are consumed and prepared in various ways. The most prevalent forms of consumption in Perú and Bolivia are “Kañihuaco” (Qañiwaku) and “pito of Kañihuaco,” respectively. It has been estimated that the production rate of this flour is between 12 and 15 kg/day [14]. The consumption of Kañihuaco has increased with the dissemination of the knowledge of its use as a superfood and in combination with other flours or inputs to produce other products. The exact current-referenced figures for Kañihuaco production are unknown [28]. Kañihuaco can be consumed alone or mixed with boiling water, milk, barley flour, fruit, or other flour to produce mazamorras (Qañiw allpi). It can also be used in soups (Qañiw soup), creams, stews, desserts, soft drinks, hot drinks, cakes, and k'ispiño or Q'uispiña. K'ispiño or Q'uispiña is a small bun made from kañihua flour and steamed, which has a consistency of biscuits or water bread and can be preserved for a relatively long time, allowing the peasant to take it on his long walks or agricultural tasks, which is not feasible with bread. Green leaves have also been prepared from stews [2,11,28,50]. Currently, kañihua seed is a necessary ingredient of designer foods as an innovative alternative to gourmet, *de novo* Andean, and vegan cuisine, among others [28,51]. Bustos et al. [52] replaced 10, 20, and 30 % of wheat flour with kañihua to prepare pasta and observed that water absorption and cooking loss increased with kañihua content, indicating a decrease in pasta quality. The Kañihua ecotype L1 showed improved cooking properties. The firmness and chewiness of the pasta decreased with the highest percentage of substitution, but the nutritional quality of the pasta improved in terms of protein content and dietary fiber. They reported that a 20 % substitution allowed for functional pasta with highly satisfactory cooking quality and adequate nutritional value. Ramirez-Lopez et al. [53] extracted starch from kañihua and quinoa seeds to evaluate their physicochemical properties and used flour from these seeds as an alternative for the development of new food formulations. They reported that both seeds present spherical shapes and regular sizes between 1.05 and 1.30 mm, the starch granules are distributed in a monomodal asymmetrical manner, and Sauter mean diameter in kañihua is < 0.961 μm compared with that of 1.099 μm in quinoa. They further reported that starches extracted from kañihua and quinoa seeds show type A polymorphism, and the amylose content was 11–14 % in kañihua and 8–12 % in quinoa. Kañihua starch showed a lower solubility (<13 %) and swelling power than recorded for quinoa starch, whereas their thermal properties, specifically retrogradation and enthalpy of gelatinization, were similar. Quispe et al. [54] evaluated the extruded mixtures of kañihua and rice in proportions of 10:90 % (w/w) and 20:80 % (w/w) and showed that the hardness of cooked kañihua–rice grains was similar to that of extruded rice and less than that of cooked brown rice. The mixtures had heterogeneous particles, and the sensory analysis results were similar to those obtained with cooked rice; thus, the extruded kañihua–rice mixture is a new food matrix with high nutritional value, optimal for consumption by rice consumers.

#### 3.6.4 Amino acid supplementation and digestibility

To evaluate the quality of a protein based on its essential amino acids, the FAO/WHO [34] recommends the use of a reference amino acid standard (PaaR) and AC. PaaR is nutritionally required by all, except those under 1 year of age. Proteins with one or more amino acids in lower quantities than those established are considered biologically incomplete and limiting, which decreases protein utilization in food [17,55]. Moreover, AC allows the estimation of the levels of limiting amino acids in fractions or percentages [40]. Thus, knowledge of PaaR and AC will allow the combination of limiting amino acids with other foods to improve amino acid complementation and quality, reflected in nutritional, physical, and mental performance [1]. Moron [56] showed that the AC of kañihua seeds comprised of 58, 16, 35, and 8.0 mg/g concentrations of Leu, Met + Cys, Phe + Tyr, and Trp, respectively, which are lower than the corresponding values of 66, 25, 63, and 11 mg/g of the amino acid pattern recommended by the FAO, respectively, limiting the amino acids that would prevent the total use of the protein. Cabieses [57] mixed kañihua seeds and panamito bean (*Phaseolus vulgaris*) in a ratio of 80:20 (w/w), kañihua and pallar (*Phaseolus lunatus* L.) in a ratio of 70:30 (w/w), and kañihua and broad bean (*Vicia faba*) in a ratio of 60:40 (w/w). They observed high amino acid complementation with these mixtures, similar to those obtained with 72 % milk casein protein. Canahua et al. [55], Bjorck and Asp [58], and Bacigalupo [59] observed that the nutritional and digestive qualities of kañihua are improved using the extrusion technique as a thermodynamic cooking and drying process, which favors the best conservation of kañihua flour and other Andean seeds. Repo-Carrasco et al. [60] evaluated the consumption of kañihua: quinoa: broad bean and drinks in a ratio of 15:75:10 (w/w/w), and the mixture showed high values of amino acid complementation, exceeding the individual consumption.

Digestibility of seeds is defined as the proportion of seeds ingested in different forms and absorbed by the body. The higher the digestibility of food, the better its absorption. Alencastre [61] calculated the digestibility of raw kañihua to be 59.2 %. This high value was attributed to the high content of soluble and dietary fibers, which could be improved through germination and cooking processes. Tapia [2] reported that the digestibility of Andean seeds was 80 %. López de Romaña et al. [62] recommended grinding and cooking kañihua seeds for >30 min to improve the digestibility indices of proteins, fats, and carbohydrates. Bock [40] evaluated the digestibility of kañihua in infants, wherein little digestion and absorption of raw and cooked whole-seed protein was observed in children under 2 years of age and recommended consuming mixes of seed flour, puree, and smoothies of fruit and milk.

#### 3.6.5 Functional and biotechnological foods

Repo-Carrasco et al. [16] reported that kañihua seeds are a critical source of total dietary fiber, lignin, and phenolic compounds with high antioxidant activity. The extruded flour of the evaluated varieties presented excellent functional properties, including the degree of gelatinization, sectional expansion index, and water solubility index. Moscoso-Mujica et al. [38] described a methodology for obtaining protein fractions from kañihua seeds of the Ramis and Cupi-Sayhua varieties, for which there was no information. They used whole wheat flour delipidized with *n*-hexane to extract all fatty acids and lipids from the seeds. Subsequently, the flour was suspended in double-distilled water in a 1:10 (w/v) ratio, stirred for 1 h at 4.0 °C, and centrifuged at 9000×*g* for 20 min at 4.0 °C, and subjected to fractionation. Albumins were extracted with a 2 N HCl solution (pH 3), 7S globulins were extracted with a solution containing 10.0 mM Na_2_HPO_4_ (pH 7.5), 1.0 mM EDTA, 100 mM NaCl, and 11S globulins were extracted with a solution containing 800 mM NaCl in phosphate buffer. Glutelins were extracted with a 0.1 M NaOH solution. They observed higher protein content in the Cupi-Sayhua variety than in the Ramis variety, with concentrations of 2.7, 4.2, 4.5, 1.7, and 4.0 g of albumins, 7S globulins, 11S globulins, prolamins, and glutelins, respectively, per 100 g of dry flour (*p* ≤ 0.05). In the electrophoretic characterization of both varieties, albumins, 7S globulins, 11S globulins, and glutelins showed bands ranging from 5 to 95, 4–55, 4–37, 6–37 kDa, respectively, with albumins showing bands of the greatest intensity. They reported that with fractionation, the protein content increased significantly in both varieties of kañihua seeds and that they are potential sources for obtaining bioactive peptides and functional ingredients in different industries. Paucar-Menacho et al. [63] evaluated replacing wheat flour in cookies with flour obtained from kañihua, kiwicha, and quinoa sprouts and antioxidant activity and the contents of phytic acid, γ-aminobutyric acid, and total soluble phenolic compounds were analyzed. They observed that kañihua and quinoa sprouts showed lower amounts of starch than in wheat sprouts but with high ash, γ-aminobutyric acid, total soluble phenolic compounds, and fat contents. Furthermore, kañihua and quinoa were nutritionally superior to wheat and kiwicha, with better bioavailability of minerals, indicating that the optimal formulation for making cookies is a combination of quinoa/kañihua. Quispe-Sánchez et al. [64] evaluated the effects of incorporating *Lepidium meyenii*, *C. pallidicaule*, *Amaranthus caudatus*, *Sesamum indicum*, and *Salvia hispanica* flours on the physical, chemical, rheological, textural, and thermal characteristics and the degree of sensory acceptance of dark chocolate tablets with 65 % cocoa. They observed that greater incorporation of flours led to increased viscosity, antioxidant concentrations, and particle size in the chocolate, but the hardness and pH decreased. Thus, the incorporation of up to 4 % flour improved the degree of acceptance of the chocolate and enhanced its antioxidant characteristics. Mérida-López et al. [4] investigated six kañihua cultivars, including Lasta Rosada, Illimani, Kullaca, and Cañawiri and ascending Saigua L24 and Saigua L25, which were decumbent owing to their stem shape and growth habits. The highest protein and ash contents were presented by whole Saigua L25 at 19.6 and 5.12 g per 100 g of dry flour, respectively, whereas the highest fat content was present in shelled Saigua L25, and the highest fiber content was present in the whole seeds of Saigua L24 at 12.5 g per 100 g of dry flour. In addition, they observed that dehulling affected the macromineral content, as opposed to microminerals, and that the growth habit influenced the C18:1 and C18:3 contents. Another research reported an oil extraction yield of 2.14 g per 100 g of the dry weight of seeds using kañihua seeds, wherein PUFAs were the most abundant with 42.1, 24.7, and 3.0 % contents of linoleic, oleic, and linolenic acids, respectively. The specific gravity was 0.897 at 20 °C, and the acid, peroxide, iodine, and saponification numbers were 0.48 mg/KOH, 5.0 mEq O_2_/kg, 175.3 g I_2_/100 g dry weight of seeds, and 1900 mg KOH/g oil, respectively. The properties used in cosmetic formulations, such as surface tension, viscosity, spreading capacity, pore size, and turbidity, are similar to those of other vegetable oils. In addition, the formulation of a cosmetic emulsion containing 5 % kañihua seed oil was stable [42].

### 3.7 Health promotion

#### 3.7.1 Research and ancestral knowledge

Ancestral knowledge of the nutraceutical use of Andean crops has allowed for greater dissemination and use in food and the prevention of many diseases [55] (Table 5). Mujica et al. [7] reported the medicinal uses of kañihua seed practiced by the Andean man from ancient times to the present day, such as in the treatment of bone fractures, dislocations, blows, and prevention of osteoporosis owing to its high calcium content, which is four times higher than that of corn and three times higher than that of rice. Due to its lithium content, it prevents melancholy and sadness. Moreover, kañihua is a lactogenic agent, and its consumption increases milk secretion in nursing mothers. Its seeds contain phytoestrogens such as daidzein and kinestein that prevent signs and symptoms of menopause and uterine cancer. It also contains betalains and betaxanthins, antioxidant compounds that preserve health, and saponins that prevent polyglobulia [7,11]. In addition, the high content of dietary fiber and PUFAs prevents and regulates cholesterol levels and prevents tuberculosis, malnutrition, and anemia owing to the high protein content with an ideal balance of essential amino acids and minerals such as iron. The orthomolecular use of Andean seeds, such as kañihua, adequately shows balanced amounts of all elements necessary for good development and growth of the human organism to prevent different diseases [28,51].

**Table 5:** Kañihua bioactives (*Chenopodium pallidicaule* Aellen) and nutraceutical/biological activities.

Bioactives	Nutraceutical/biological activities	References
Flavonoids, phenolic acids, and polyphenols. Total phenolic//Betalains and betaxanthins//Catechin, gallato, vanillic acid, kaempferol, ferulic acid, quercetin, resorcinol, and 4-methylresorcinol. Total phenolic compounds//Peptides of 3–11 amino acids//perigonium.	Antioxidant	28, 69//7//30//70//72
Total fiber, Soluble fiber, Insoluble fiber.	Constipation, water retention in biological processes, sensitization to fermentation, inhibition of digestive enzymes, facilitating the binding of bile acids, reducing plasma cholesterol levels, increasing intestinal peristalsis, and body temperature control.	38, 39
linoleic acid, linolenic acid, oleic acid, unsaturated fatty acids (PUFAs).	Nutrition, caloric activity, regulation of blood pressure, coagulation, kidney function, precursors of prostaglandins, leukotrienes, and prostacyclins, regulates cholesterol levels.	37, 40, 14, 11.
Calcium, magnesium, and iron.	Co-factors in metabolic pathways.	39, 41
Vitamin K, C, and tocopherol isomers.	Antioxidant activity that provide a greater extent of conservation to the seed.	37, 42
Iron, high protein content, optimal balance of essential amino acids, and minerals.	Antianemic.	65, 66
Lithium.	Prevents melancholy and sadness.	7, 26, 49
Phytoestrogens (daidzein and kinestein).	Prevent signs and symptoms of menopause and uterine cancer.	7, 26, 49
Protein content, protein fractions: albumins, 7S globulins, 11S globulins and glutelins; essential amino acids.	Malnutrition, antianemic, prevents tuberculosis.	31, 65, 66
Granulated starch.	Input or component for pharmaceutical, cosmetic, chemical, and food industries.	47
Total phenolic, quercetin, gallic acid, rutin, and chlorogenic acid//Peptides of 3–11 amino acids.	Inhibition of α-glucosidase and α-amylase (antidiabetic and antihypertensive for inhibition in ACE-I)//ACE-I inhibition.	69, 74//70
Calcium.	Treatment of bone fractures, dislocations, blows, and prevention of osteoporosis.	7,
Saponins.	Polyglobulia.	7,11
Antimicrobial peptides (PAMs): Glob 7S KR 9h (1:10), Glob 11S KS 2h (1:50), Glut KS 2h (1:10), Glut KS 4h (1:10).	Antimicrobial activity (*Escherichia coli*, *Staphylococcus aureus*), antifungal activity.	31

#### 3.7.2 Nutraceuticals

##### 3.7.2.1 Anemia-based studies

Novak et al. [65] evaluated 25 non-pregnant and non-lactating women of poor rural origin with a 35 % prevalence of anemia from the Peruvian Altiplano at 3850 m of altitude. Kañihua flour with vitamin C was administered orally every 24 h in a 50:100 g (w/w) ratio for 7 weeks, and hemoglobin levels were evaluated weekly. They observed that post-treatment, the hemoglobin levels of women in the kañihua treatment group were higher than that of the women in the control group (p ≤ 0.05). Moreover, the hemoglobin values reached normal levels corresponding to a high-altitude population. The authors recommend daily consumption of kañihua with vitamin C at the evaluated dose as an adequate source of iron and protein to prevent mild anemia. Moscoso-Mujica et al. [66] evaluated the antianemic activity of extruded flour obtained from the Negra Collana variety of quinoa and the Ramis variety of kañihua owing to their high protein content and optimal balance of essential amino acids and minerals. The results revealed that the protein contents in quinoa Negra Collana was 22.0 ± 0.1 %, whereas it was 16.2 ± 0.1 % in kañihua Ramis. The fat content in kañihua was 10 %, higher than that of quinoa at 3.5 %. The fiber contents in quinoa and kañihua were 7.1 and 3.8 %, respectively, which are higher than that in cereals, whereas the moisture, ash, fat, and carbohydrate contents of quinoa and kañihua flour showed values comparable to that in cereals [67]. In addition, the extruded flour of the quinoa variety Negra Collana contained 19.8 mg of iron, and the extruded flour of the kañihua variety Ramis contained 17.6 mg. In the acute toxicity test in rats, consumption of both flours was considered safe up to a dose of 15000 mg/kg [68], confirmed by anatomopathological observation of organs such as the liver, stomach, lungs, kidneys, and brain. There was no inflammation or internal bleeding in any of the organs. None of the evaluated dosages showed morphophysiological alterations and preserved the diameter and characteristics of each organ compared with that in the control. In the evaluation of antianemic activity, the basal average of 29.3 ± 0.2 % of hematocrit was observed in the group of anemic rats treated with quinoa flour, and in 12 weeks, it increased to 53.8 ± 0.3 % (*p* ≤ 0.05). The group of anemic rats treated with kañihua flour showed a baseline average of 29.5 ± 0.3 % of hematocrit, and in 12 weeks, it increased to 51.7 ± 0.3 % (*p* ≤ 0.05). A group of rats without anemia treated with quinoa and kañihua flour showed a basal average of 50.2 ± 0.2 % and 49.3 ± 0.3 %, and in 12 weeks, it increased to 55.2 ± 0.2 % and 54.8 ± 0.1 %, respectively. It was concluded that the oral administration of 360 mg/kg of quinoa flour and kañihua flour every 24 h increased hematocrit levels by 24.5 ± 0.5 % and 22.2 ± 0.3 %, with the rats showing average weights of 65.8 ± 0.3 g and 59.2 ± 0.1 g and mean heights of 6.8 ± 0.1 cm and 5.7 ± 0.5 cm, respectively (*p* ≤ 0.05).

##### 3.7.2.2 Studies on diabetes, Angiotensin-I-Converting enzyme inhibitors and antioxidant activity

Ranilla et al. [69] showed the functionality of thermally processed Peruvian Andean seeds by evaluating the inhibition of α-glucosidase and α-amylase enzymes that participate in the early stages of type 2 diabetes and that of the Angiotensin-I-Cconverting enzyme (ACE-I) that participates in arterial hypertension. In addition, these seeds were analyzed for their total phenolic content and phenolic and antioxidant profiles. They observed that the purple corn (*Zea mays* L.) cereal exhibited high antioxidant activity linked to the elimination of 77 % of free radicals with the highest total phenolic content of 8.0 ± 1.0 mEq gallic acid/g weight and α-glucosidase inhibitory activity of 51 %. Quinoa and kañihua showed high quercetin levels of 1131 ± 56 and 943 ± 35 μg aglycone/g weight, with higher antioxidant activity of 86 and 75 %, respectively. The legume tarwi (*L. mutabilis*) inhibited 52 % of ACE-I activity at a concentration of 5 mg/g weight. α-amylase inhibition was not observed with any sample. In addition, they indicated that a combination of Peruvian Andean seeds could be used to develop effective dietary strategies to prevent type 2 diabetes and hypertension. Chirinos et al. [70] evaluated in vitro hydrolysates and peptides obtained from kañihua seeds. The hydrolysate obtained with Neutrase–Alcalase treatment after 180 min of digestion at 50 °C showed improved antioxidant activity of 2.12 μmol Trolox equivalent (TE)/mg and ACE-I inhibition of 69.8 %. After purification, fraction III showed peptides of 3–11 amino acids with antioxidant activity of 3.18 μmol TE/mg and ACE-I inhibition of 78.4 %. Coronado-Olano et al. [71] identified gallic acid, rutin, and chlorogenic acid in kañihua seeds while evaluating the inhibition of carbohydrate hydrolyzing enzymes associated with type 2 diabetes, such as α-amylase with the half-maximal inhibitory concentration (IC_50_) of 7.99–34.05 μg/mL and α-glucosidase with IC_50_ of 8.07–11.58 μg/mL. They reported that total phenolics, flavonoids, gallic acid, and chlorogenic acid participated significantly in α-glucosidase enzyme inhibition. Callohuanca-Pariapaza et al. [72] evaluated the color of the perigonium and the antioxidant capacity of four accessions of *C. pallidicaule* with perigonia of defined colors such as light yellow, orange, purple, and black. They observed an antioxidant capacity equivalent to that of Trolox, which varied based on different color intensities of the perigonium (*p* ≤ 0.05). Black-colored perigonia showed a value of 5 g eq. Trolox/100 g sample, higher than that exhibited by perigonia of other colors. Thus, flavonoid content correlated directly with the color intensity of the perigonium, which exhibited antioxidant capacity. Serena-Romero et al. [73] (2023) reported the in vitro protein digestibility of amaranth and kañihua at 79.19 and 71.22 %, respectively. At a concentration of 0.750 mg/mL, simulated gastrointestinal digestion improved cellular antioxidant activity, and peptides from kañihua fractions >5 kDa were the main contributors to cellular antioxidant activity in human intestinal Caco-2 and hepatic Hep-G2 cell lines, with activities of 85.55 and 82.57 %, respectively. Thus, the hydrolysates of amaranth and kañihua showed high digestibility, and their peptides exhibited powerful antioxidant properties that are important for health.

##### 3.7.2.3 Antimicrobial research

Moscoso-Mujica et al. [33] obtained antimicrobial peptides (PAMs) from the protein fractions of the Ramis and Cupi-Sayhua varieties of kañihua with a final concentration of 1 % (w/v) at different enzymatic hydrolysis times based on the protein fraction and two different enzyme: substrate ratios of 1:10 and 1:50 for hydrolysis. They showed that of the 216 hydrolysates, those obtained in simulated pepsin–pancreatin digestion *in vitro* showed a higher degree of hydrolysis (DH) of 7 and 67 % compared with the DH of 13 and 54 % for those obtained using Alcalase, respectively. It was observed that only 28 hydrolysates presented inhibition percentage (PI) ≥ 45 % for *Escherichia coli*, *Staphylococcus aureus*, and *Candida albicans* (*p* ≤ 0.05) compared with that in the respective controls. Four promising peptides were purified, which showed high resolution and increased antimicrobial activity, most significantly with a high degree of purification and sizes of 6.5 kDa visualized electrophoretically. Thus, Glob 7S KR 9h (1:10) showed PI of 54, 52, and 19 % for *E. coli*, *S. aureus*, and *C. albicans*, respectively, whereas Glob 11S KS 2h (1:50) showed PI of 75, 47, and 33 % for *S. aureus*, *E. coli*, and *C. albicans*, respectively. Glut KS 2h (1:10) showed PI of 79, 56, and 41 % for *E. coli*, *C. albicans*, and *S. aureus*, whereas Glut KS 4h (1:10) showed PI of 70 and 52 % for *C. albicans* and *S. aureus*, respectively, and showed minimum inhibitory concentration of 95 % for *E. coli* (*p* ≤ 0.05). Three of these peptides were cationic, whereas one was anionic. The hydrolysates and PAMs of both kañihua varieties are natural alternatives for treating infections caused by microorganisms resistant to current antibiotics and can be used as biopreservatives and additives for processed foods and nutraceuticals in the food industry. Kim and Lida [74] developed an older-friendly kañihua food conforming to Korean industrial standards. They analyzed the nutrient composition and physiological activity of this kañihua food and the concentration of gelling agents, such as guar gum, locust bean gum, and xanthan gum, in this food. They observed that kañihua had a suitable composition of fatty acids and exhibited antioxidant and antidiabetic properties. To obtain the hardness necessary to chew kañihua food with the tongue, guar gum, carob gum, and xanthan gum must be added at concentrations less than 1.97, 4.03, and 8.59 %, respectively.

### 3.8 Conclusions

Kañihua thrives in wild and unpredictable areas of the highlands of Perú and Bolivia and is resistant to frost, drought, saline soils, and pests. It presents great genetic diversity. The seeds contain low levels of saponins with a bitter taste, which allows for rapid and cheap production of edible flour using kañihua. Despite its cultivation since pre-Hispanic times, the cultivated area has been decreasing, leading to an uncertain future of cultivation; however, in recent years, valuable innovations have enables substantial advances in production, transformation, marketing, use, nutrition, feeding, conservation of genetic resources, and gastronomy, with the promotion of cultivation, consumption, and research. As a food with high nutritional value, it contains essential amino acids, such as Thr, Ile, and Lys, in higher quantities than that in quinoa, kiwicha, rice, and wheat, and Val compared with that in quinoa and kiwicha. In addition, it contains unsaturated fatty acids with omega-3, -6, and -9 fatty acids and a range of minerals and vitamins, including iron, that participate in the vital functions of an organism. It is a natural source of soluble fiber and carbohydrates. In addition, it has wide nutritional, culinary, industrial, and nutraceutical uses and is considered a reliable source of human and fodder food and a security backup when other crops fail. Kañihua seeds in different forms such as total protein and protein fractions (albumins, globulins, and glutelins), are potential industrial sources of functional foods such as Kañihuaco, Llipta, energy bars, breads, noodles, cookies, cakes, culinary ingredients, innovative alternative to gourmet, *de novo* Andean, vegan cuisine, gastronomic garnishes, natural food preservatives, biofilms, and among others. Also, nutraceuticals such as antimicrobial and antifungal peptides (Glob 7S KR 9h (1:10), Glob 11S KS 2h (1:50), Glut KS 2h (1:10), Glut KS 4h (1:10), celiac disease, antioxidant, antihypertensive and antidiabetic peptides, which can be used as preventive and curative medicine in public health. Thus, the medicinal properties of kañihua highlight the importance of consuming this Andean seed, which in addition to feeding and nourishing, helps prevent different diseases of high incidence worldwide and improve food security throughout the Andean region and the world.

## Ethics statement

Review and approval by an ethics committee were not required for this study because it is a literature review, and no new data were collected and analyzed. Informed consent was not required for this reason.

## Data availability statement

No data was used for the research described in the article.

## Additional information

No additional information is available for this paper.

## Funding and acknowledgments

This research was supported by the 10.13039/501100008786Universidad Nacional Mayor de San Marcos RR N° 004305-2024-R/UNMSM and project number A24041131.

## CRediT authorship contribution statement

**Gladys Moscoso-Mujica:** Writing – review & editing, Validation, Supervision, Resources, Project administration, Methodology, Investigation, Funding acquisition, Formal analysis, Data curation, Conceptualization. **Ángel Mujica:** Writing – review & editing, Methodology, Conceptualization. **Ernesto Chura:** Writing – review & editing. **Noelia Begazo:** Writing – review & editing. **Karin Jayo-Silva:** Writing – review & editing. **Marcos Oliva:** Formal analysis, Data curation.

## Declaration of competing interest

The authors declare that they have no known competing financial interests or personal relationships that could have appeared to influence the work reported in this paper.
